# Hydrate-Based Methane Storage in Biodegradable Hydrogels Absorbing Dilute Sodium P-Styrenesulfonate Solution

**DOI:** 10.3390/gels11010001

**Published:** 2024-12-24

**Authors:** Fangzheng Hua, Kang Tan, Jingyu Lv, Fei Wang, Mengting Sun

**Affiliations:** Shandong Engineering Laboratory for Preparation and Application of High-Performance Carbon-Materials, College of Electromechanical Engineering, Qingdao University of Science & Technology, Qingdao 266061, China; my1767267350@163.com (F.H.); 19937601544@163.com (K.T.); 15054823867@163.com (J.L.)

**Keywords:** methane hydrates, eco-friendly hydrogels, trace SS, synergistic promotion, recyclability

## Abstract

Developing an exceptional reaction medium with high promotion efficiency, desirable biodegradability and good recyclability is necessary for hydrate-based methane storage. In this work, a kind of eco-friendly hydrogel, polyvinyl alcohol-co-acrylic acid (PVA-co-PAA), was utilized to absorb dilute sodium p-styrenesulfonate (SS) solution, for constructing a hybrid reaction medium for methane hydrate formation. Hydrogels or dilute SS solutions (1–4 mmol L^−1^) had weak or even no promoting effects on hydrate formation kinetics, while the combination of them could synergistically promote methane hydrate formation. In hydrogel-SS hybrid media containing 1, 2, 3 and 4 mmol L^−1^ of SS solutions, the storage capacity reached 121.2 ± 1.6, 121.5 ± 3.1, 122.6 ± 1.9 and 120.6 ± 1.6 *v*/*v*, respectively. In this binary reaction system, the large surface area of hydrogels provided hydrate formation with sufficient nucleation sites and an enlarged gas–liquid interface, and in the meantime, the dilute SS solution produced an adequate capillary effect, which together enhanced mass transfer and accelerated hydrate formation kinetics. Additionally, the hybrid medium could relieve wall-climbing hydrate growth and improve poor hydrate compactness resulting from the bulk SS promoter. Moreover, the hybrid medium exhibited a preferable recyclability and could be reused at least 10 times. Therefore, the hydrogel-SS hybrid medium can serve as an effective and eco-friendly packing medium for methane hydrate storage tanks, which holds great application potential in hydrate-based methane storage technology.

## 1. Introduction

Natural gas, mainly comprising methane (>80%), is a critical clean energy source and has been continuously increasing in global consumption in the past few decades [1,2]. Although natural gas is characteristic of high energy density and low carbon emission, it presents in a gaseous state under normal conditions, which brings the undesirable result that it is difficult to be stored and transported efficiently. This issue constrains the utilization of natural gas [3,4].

Currently, available technologies for natural gas storage and transportation include compressed natural gas (CNG) and liquefied natural gas (LNG) [5]. Among them, LNG can achieve a remarkable natural gas storage capacity over 600 *v*/*v*, and is accordingly the most accessible approach. However, an extremely low temperature (e.g., 111 K) is necessary for LNG [6,7]. An alternative technology based on natural gas hydrates (NGHs), termed solid natural gas (SNG), provides a promising solution for natural gas storage and transportation. NGHs are ice-like clathrate crystalline compounds consisting of natural gas and water at low temperatures and high pressures, and in this case, natural gas molecules are enwrapped in lattices formed by hydrogen-bonded water molecules. SNG can achieve a high storage capacity (~170 *v*/*v*), and more importantly, NGHs can be preserved under atmospheric pressure at a much higher temperature (e.g., 268.15 K) compared to LNG [3,8,9,10,11]. However, NGH formation is an interfacial process between gas and water, and a thin solid hydrate film will be formed and seal off the gas–liquid interface, which hinders contact between gas and water and causes weak mass transfer. Consequently, NGH formation kinetics are naturally slow, which seriously restricts applications of SNG technology [12].

Tremendous efforts have been made to improve the mass transfer of NGH formation. Mechanical methods (e.g., stirring [13] and gas bubbling [14]) continuously break the solid hydrate film on the gas–liquid interface for enhancing mass transfer. One problem is that the frictional heat produced by mechanical operation negatively impacts the exothermic hydrate formation, and another problem is that the gradually increasing solid hydrate blocks will hinder or even cease mechanical operation. Moreover, mechanical operation needs extra energy consumption and equipment costs [15]. Kinetic promoters are a sort of additive that are capable of increasing the mass transfer of NGH formation. Surfactants, e.g., sodium dodecyl sulfate (SDS) [16], are the most effective kinetic promoters. However, abuses of them will pose chemical pollution risks to the ecosystem, and meanwhile, surfactants usually generate a bulk of foam when hydrates dissociate, which is unfavorable to recycling use during field applications [17]. Another class of kinetic promoters are porous media, e.g., active carbon [18], metal-organic frameworks [19] and silica sand [20], and they can provide abundant sites for hydrate nucleation and increase mass transfer of hydrate growth due to their high porosities, which have the potential to be packing media for portable NGH storage tanks. However, hydrates usually grow out of pores, and the dissociated water cannot be re-absorbed by porous media during recycling use, which also results in poor recyclability [21]. Therefore, developing a packing medium that has excellent promotion efficiency as well favorable recyclability is essential for the feasibility and practicability of SNG technology.

Hydrogels are lightly cross-linked polymers with 3D networks, and accordingly have strong water absorption and retention capabilities [22,23]. In the past few years, several types of hydrogels, e.g., poly(2-hydroxyethyl methacrylate) (PHEMA) [24,25], poly(styrenesulfonate-co-acrylamide) (PSS-co-AAm) [26,27] and polyvinyl alcohol-co-acrylic acid (PVA-co-PAA) [28,29], have been regarded as novel reaction media for NGH formation. Although these hydrogels exhibit promoting effects on methane hydrate formation kinetics without mechanical stirring, all of them are more or less flawed. PHEMA hydrogels could not function well by themselves, and just reached the dreadful methane storage capacity ranging 20–70 *v*/*v*; if they were combined with dry water, this hybrid system could markedly accelerate methane hydrate formation [24]. PSS-co-AAm hydrogels performed excellently in promoting methane hydrate formation, and reached a methane storage capacity of 110.84 ± 1.01 *v*/*v*; however, due to the weak mechanical property of polymeric frameworks, PSS-co-AAm showed an awful recyclability and could be reused just two times [26]. PVA-co-PAA hydrogels, which are biodegradable and hold desirable mechanical properties, could be reused at least seven times; however, when they were used alone, the promotion efficiency was much lower and they could achieve a methane storage capacity of just 75.17 ± 4.66 *v*/*v* [28]. Furthermore, PVA-co-PAA hydrogels were employed as carriers for the trace SDS solution (0.1–0.4 mmol L^−1^), and this binary reaction medium attained a higher methane storage capacity of 120.62 ± 1.57 *v*/*v*; meanwhile, it could be reused at least eight times [29]. Inspired by previous studies, in this work, PVA-co-PAA hydrogels were used to absorb dilute sodium p-styrenesulfonate (SS) solution (1–4 mmol L^−1^), for preparing a binary reaction medium for methane hydrate formation. The hydrate formation kinetics, morphological observation, microscopic hydrate distribution and recyclability of this hybrid medium were systematically investigated.

## 2. Results and Discussion

### 2.1. Methane Hydrate Formation Kinetics in Hydrogel-SS Hybrid Medium

**Figure 1:**
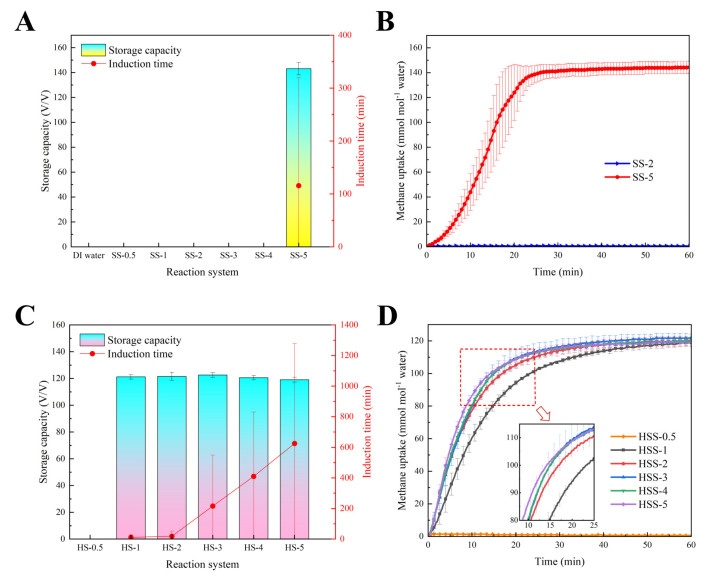
Storage capacities, induction times (**A**) and curves of methane uptake over time (**B**) in the presence of bulk distilled water or SS solutions; storage capacities, induction times (**C**) and curves of methane uptake over time (**D**) when using hydrogel-SS hybrid media for hydrate formations.

**Figure 2:**
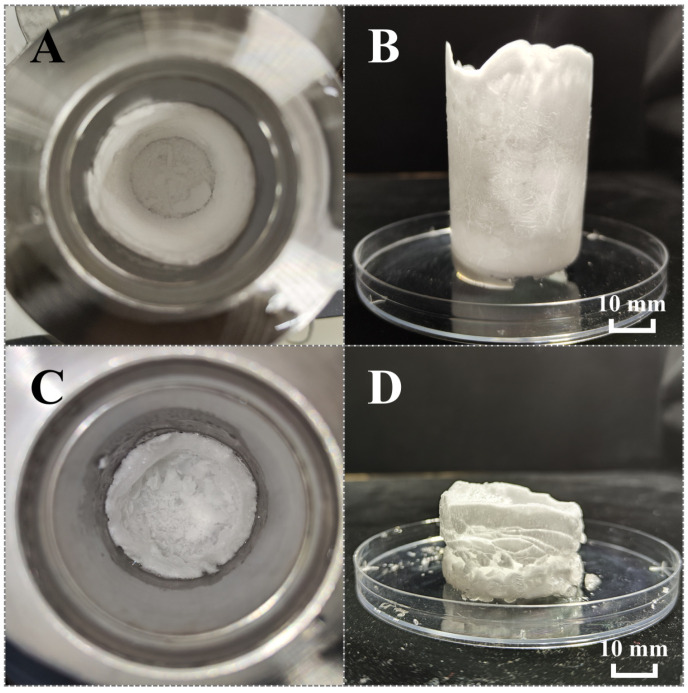
Appearances of hydrates formed in stainless steel reactor in SS-5 (**A**) and (**B**) or HSS-2 (**C**) and (**D**) group.

Figure 1A,B present the methane hydrate formation kinetics in bulk water and dilute SS solutions. In DI water without additives or mechanical stirring, the obvious pressure drop accompanied by a temperature rise failed to be detected during the whole experimental period, and the methane uptake was approximately 0 at each test time point, indicating that hydrate formation did not occur on this occasion. Methane inherently has low solubility in water; meanwhile, methane hydrate formation is an interfacial process between gas and water, a thin hydrate film would form and seal off the gas–liquid interface, further intensifying hindrance on mass transfer and ceasing continuous hydrate formation. When using bulk SS solutions as reaction systems, it was found that bulk SS solutions at concentrations of 0.5–4 mmol L^−1^ (SS-0.5 to SS-4 group) still could not induce continuous hydrate formation, which was the same in the DI water group. When the SS concentration rose to 5 mmol L^−1^ (SS-5 group), a remarkable promotion was observed; the induction time, HGR_t90_, methane uptake and storage capacity were 115.8 ± 203.8 min, 0.380 ± 0.075 mmol mL^−1^ min^−1^, 144.4 ± 4.9 mmol mol^−1^ and 143.1 ± 4.9 *v*/*v*, respectively. Results showed that the SS solution under a low concentration equal to or lower than 4 mmol L^−1^ had no promoting effects at all on methane hydrate formation, whereas the bulk SS solution at 5 mmol L^−1^ could markedly accelerate hydrate formation. It is worth noticing that wall-climbing growth was observed in the SS-5 group (Figure 2), which was similar to the methane hydrate formation promoted by SDS [30] or some amino acids [31]. From this phenomenon, it was deduced that 5 mmol L^−1^ of SS could generate a strong capillary effect that enabled hydrates to rapidly grow along the reactor sidewall and form as hollow cylinder-shaped layers. This might be the main reason for the promotion performance, and needs to be verified in the following morphological observation. Accordingly, 5 mmol L^−1^ of SS could be deemed as the concentration threshold for promoting methane hydrate formation, and a concentration lower than 5 mmol L^−1^ could be regarded as a dilute solution. However, this dosage is relatively high compared to 1 mmol L^−1^ of SDS, which has outstanding promotion performance [28]. As a non-biodegradable chemical reagent, the abuse of SS can cause severe pollution and chronic toxicity in the ecosystem. Reducing the dosage is a benefit for the utilization of the SS promoter and is much more eco-friendly.

To activate the promotion potentials of dilute SS solutions (0.5–4 mmol L^−1^), biodegradable PVA-co-PAA hydrogels (with a BOD_5_/COD value of 0.324 ± 0.008) were utilized as carriers for absorbing dilute SS solutions to prepare novel hybrid reaction media for methane hydrate formation. Figure 1C,D illustrate the methane hydrate formation kinetics in hydrogels absorbing different concentrations of SS solutions. The HSS-0.5 group, containing hydrogels absorbing 0.5 mmol L^−1^ of SS, still failed to induce a hydrate formation reaction, implying that the combination of them had no obvious promoting effects on hydrate formation. When the concentration continuously increased, the hybrid reaction medium started to play a part in accelerating hydrate formation. The HSS-1, HSS-2, HSS-3, HSS-4 and HSS-5 groups, which referred to hydrogels absorbing SS solutions at 1, 2, 3, 4 and 5 mmol L^−1^, respectively, all could trigger vigorous hydrate formation. As listed in Table 1, the induction periods of the HSS-1, HSS-2, HSS-3, HSS-4 and HSS-5 groups were 11.3 ± 16.3, 18.7 ± 32.3, 216.0 ± 332.6, 410.0 ± 420.4 and 625.3 ± 653.2 min, respectively. Evidently, the induction time was shortened along with the elevated SS concentration. According to the measurement of absorption capability, the absorption capacities of PVA-co-PAA hydrogels on 1, 2, 3, 4 and 5 mmol L^−1^ of SS solutions were 251.9 ± 6.2, 203.9 ± 2.3, 189.3 ± 3.1, 175.5 ± 5.6 and 151.1 ± 9.7 g g^−1^, respectively (Table 2). Along with the growing SS concentration, the salt ion concentration therewith increased, leading to the reduced absorption capability of hydrogels. With a lower absorption capacity, there might be more free water on the surfaces of hydrogel particles, which would cause hydrogel agglomeration and a narrower surface area. Consequently, there were fewer nucleation sites for hydrate formation, resulting in a prolonged induction time. The HGR_St90_ of HSS-1, HSS-2, HSS-3, HSS-4 and HSS-5 was 0.184 ± 0.006, 0.250 ± 0.004, 0.296 ± 0.053, 0.292 ± 0.021 and 0.306 ± 0.023 mmol mL^−1^ min^−1^, respectively, and this showed that the hydrate growth rate was gradually quickened with the increased SS concentration. With a higher SS concentration, stronger promoting effects might be generated, bringing about a quicker hydrate growth rate. The storage capacities of HSS-1, HSS-2, HSS-3, HSS-4 and HSS-5 were 121.2 ± 1.6, 121.5 ± 3.1, 122.6 ± 1.9, 120.6 ± 1.6 and 119.1 ± 1.8 *v*/*v*, respectively. Results showcased that the storage capacities of the hybrid reaction media maintained at approximately 120 *v*/*v*; the value was first slightly increased and then decreased, and reached the highest value of 122.6 ± 1.9 *v*/*v* in the HSS-3 group with a moderate SS concentration of 3 mmol L^−1^. Accordingly, when using hydrogels to absorb dilute SS solutions at concentrations of 0.5–4 mmol L^−1^, robust hydrate growth can be achieved, and comparatively, the unabsorbed bulk SS solutions at the same concentrations have no promoting effects, which convincingly proves that hydrogels and dilute SS solution can synergistically improve methane hydrate formation kinetics. In hydrogel-SS hybrid reaction medium, on the one hand, hydrogel particles exhibit a large surface area, which not only provides sufficient nucleation sites for hydrates, but also increases mass transfer because of an enlarged gas–liquid interface; on the other hand, the absorbed SS produce a strong capillary effect, which further enhances mass transfer and accelerate growth rate. Although hydrogels and dilute SS solution can achieve a wonderful synergistic promotion, their storage capacity is not high enough, only ~120 *v*/*v* in this work and relatively lower than the 143.1 ± 4.9 *v*/*v* of 5 mmol L^−1^ of the bulk SS solution (SS-5). This is because hydrogels have a strong absorption capability, and they can tightly lock some water inside of their polymer networks and prevent these water molecules from participating in hydrate formation, which causes poorer water-to-hydrate conversion and a lower storage capacity. Furthermore, the promotion efficiency of HSS-5 was revealed to be weaker than that of SS-5, meaning that hydrogels might play an antagonistic role when the concentration is above the threshold value.

### 2.2. Morphological Evolutions of Hydrate Formation and Dissociation

**Figure 3:**
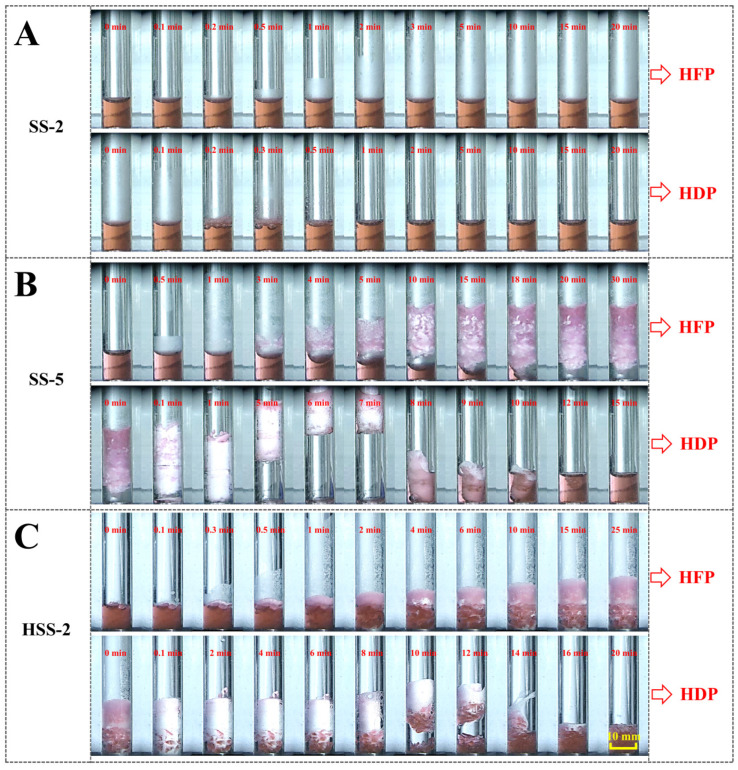
Morphological evolution of methane hydrate formation and dissociation in the presence of 2 mmol L^−1^ SS solution (**A**), 5 mmol L^−1^ SS solution (**B**) and the mixed medium of hydrogel and 2 mmol L^−1^ SS solution (**C**).

To unveil the mechanism of synergistic promotion achieved by hydrogels and dilute SS solutions, morphology observations on hydrate evolutions during formation and dissociation processes in hydrogel-SS hybrid medium were performed. Figure 3 shows the hydrate formation and dissociation processes in the SS-2, HSS-2 and SS-5 systems. In the SS-2 system, there was no vigorous hydrate formation, and only thin hydrate films formed on the horizontal gas–liquid interface as well as the glass wall above the liquid level; and then these hydrate films dissociated when the reactor was depressurized, which caused slight fluctuations in the liquid level (Figure 3A). It could be inferred that the mass transfer was still dreadful in 2 mmol L^−1^ of SS solution. When the concentration went up to 5 mmol L^−1^, robust hydrate formation took place. In this case, hydrate nucleation initially occurred on the horizontal gas–liquid interface in less than 0.5 min, and then the hydrates violently grew in both upward and downward directions along with the glass sidewall in a very short duration of about 20 min, and ultimately formed as hollow cylindrical layers that closely attached to the inside wall of the glass reactor. Figure 3B visually showcases that the water in the bulk solution could be sucked to reaction sites of hydrate formation, leading to rapid and continuous hydrate formation. Results convincingly demonstrated that 5 mmol L^−1^ of SS could generate an adequate capillary effect, which dramatically improved mass transfer and promoted hydrate formation kinetics. Notably, the hydrate height was markedly increased after the hydrate formation process (HFP) compared with the initial solution height, meaning that SS-promoted hydrates might suffer from a low apparent density and poor compactness. Although 5 mmol L^−1^ of SS has a distinguished promotion efficiency, the poor hydrate compactness might be a negative factor, especially during large-scale field applications. Figure 3C shows that vibrant hydrate formation was also achieved in the HSS-2 system. Initially, hydrate nucleation occurred on the top surface of stacked hydrogels in less than 0.1 min, suggesting that there might be an extremely short induction time for the nucleation period. Hydrogel particles played a critical role in facilitating hydrate nucleation due to their large surface area, which provided plenty of nucleation sites and increased mass transfer. Afterwards, the wall-climbing hydrate growth started, and the hydrates rapidly grew upward from the gas/liquid/solid interline along the reactor sidewall, which appeared as frosted layers (in white color) attached to the reactor sidewall. At the same time, the height of stacked hydrogels in the pink color lowered, implying that some water ran out of hydrogels to participate in hydrate formation, which caused the volume shrinkage of hydrogels. During the hydrate growth period, the capillary effect generated by SS might be the primary cause for improved mass transfer. Compared with SS-5, the height increment of hydrates in HSS-2 was much smaller. A parameter, the elongation coefficient (EC), was used to reflect the hydrate compactness formed in SS-5 and HSS-2 systems. As presented in Table 3, the ECs of SS-5 and HSS-2 were 1.847 and 1.109, respectively, indicating that the hydrate compactness of the hydrogel-SS hybrid medium was superior to that of 5 mmol L^−1^ of the bulk SS solution. In the hybrid medium, the strong water retention capability of hydrogels restrained the upward movement of water along the reactor sidewall, and meanwhile, the large surface area of hydrogel particles could replace the reactor sidewall to serve as climbing surfaces for hydrate growth, which could further relieve the climbing height of hydrates on the reactor sidewall. Although the promotion efficiency of HSS-2 was relatively inferior to SS-5, it contained a low dosage of SS and could induce the formation of more compact hydrates, suggesting that the hydrogel-SS hybrid medium might perform better on environmental protection and hydrate compactness improvement.

**Figure 4:**
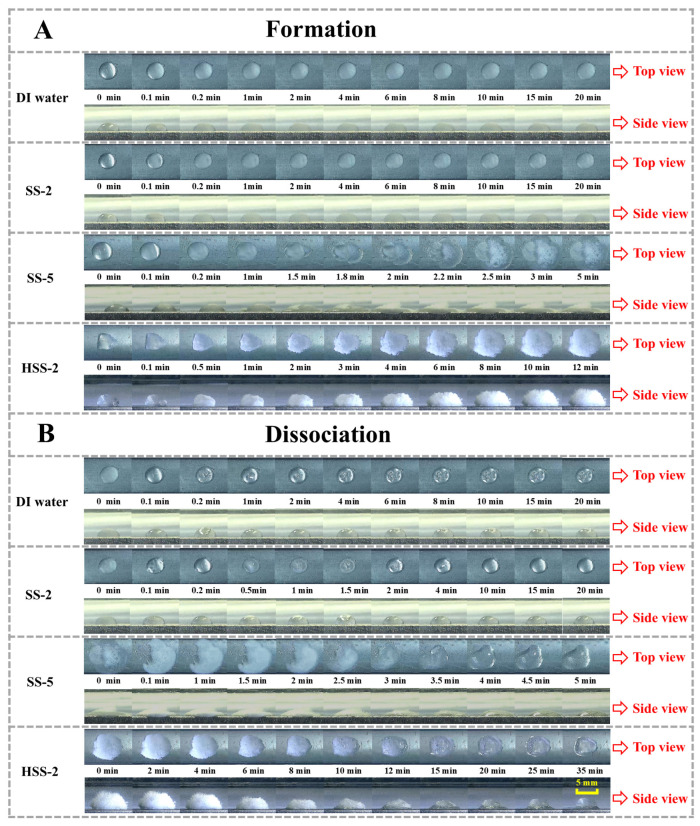
Morphological evolutions of methane hydrate formation (**A**) and dissociation (**B**) from a single-grained hydrogel or SS droplets.

To observe the morphological evolutions of hydrates formed in hybrid medium more microscopically, methane hydrate formation and dissociation from a single-grained hydrogel absorbing 2 mmol L^−1^ of SS solution were observed by an optical microscope. As displayed in Figure 4A, in the HSS-2 group, vigorous hydrate formation was observed: hydrates initially nucleated at the surface of hydrogel particles, making the hydrogel surface turn white-frosted; afterwards, from 1 to 10 min, the absorbed water rapidly transferred out of the hydrogel particle and was involved in hydrate growth, forming as a white hairy hydrate particle with a larger volume compared to the original hydrogel particle. Comparatively, both distilled water (DI water group) and 2 mmol L^−1^ of bulk SS solution (SS-2 group) were subjected to terrible mass transfers, and as a result, only a thin frosted hydrate shell was formed, which sealed off the droplet surface. Results showcased that the presence of the hydrogel particle could unlock the promotion potential of 2 mmol L^−1^ of SS solution, and the combination of them, mainly profiting from the large surface area of hydrogels and capillary effect of SS, could synergistically achieve rapid hydrate formation. In the SS-5 group, water in droplets violently extended along the plate surface, and reacted with methane gas to form hydrate layers covering a large area of the plate. The climbing hydrate growth on the plate also confirmed the existence of a strong capillary effect; however, this growth pattern resulted in a large occupation area of hydrates that referred to poor hydrate compactness. Moreover, during the hydrate dissociation process (HDP), the migratory water could not move back to the original location, and consequently, the droplet could not recover to its original shape before hydrate formation. As for HDP in the HSS-2 group, hydrate dissociation caused the white hairy hydrate particle to become smaller, and then the encased hydrogel particle was exposed. Almost all water released from hydrate dissociation was re-absorbed by the hydrogel particle, making the hydrogel particle recover to the original shape and volume (Figure 4B). This is extremely different from conventional porous media, e.g., active carbon [19] and silica sand, which scarcely re-absorb the dissociated water out of pores because they lack strong absorption and retention capabilities. Accordingly, the strong absorption performance of hydrogels may perhaps endow them with desirable recyclability when they are used as a reaction medium for hydrate formation, and therefore, hydrogels are expected to be a favorable packing medium for hydrate-based methane storage.

**Figure 5:**
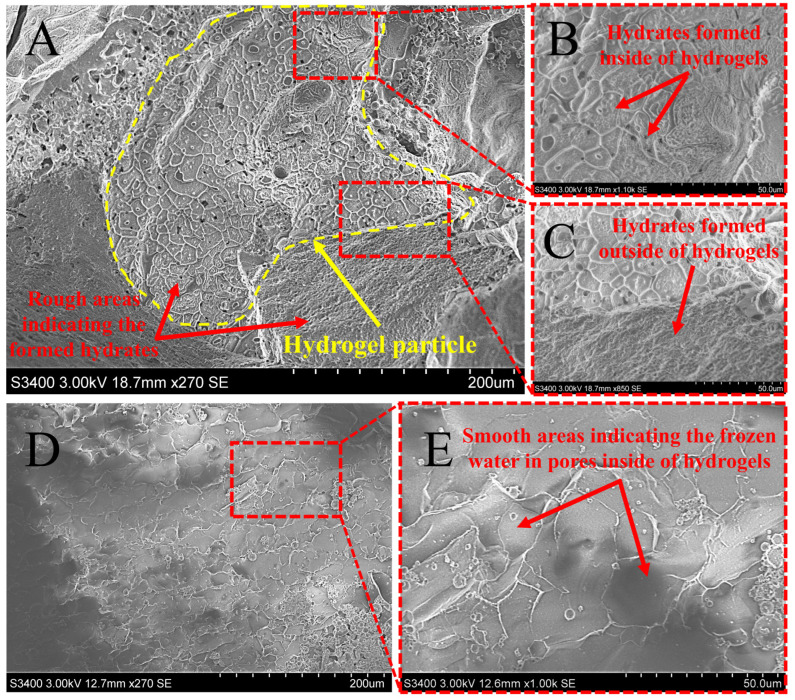
Cryo-EM images: (**A**–**C**): methane hydrates formed in hydrogels absorbing 2 mmol L^−1^ of SS solution (the region inside of yellow line is a whole hydrogel particle); (**D**,**E**): frozen hydrogels absorbing 2 mmol L^−1^ of SS solution pretreated at 253.15 K for 24 h.

The hydrate distribution in the hydrogel-SS hybrid medium was observed by Cryo-EM. Figure 5A–C reveal the methane hydrates formed in hydrogels absorbing 2 mmol L^−1^ of SS solution, while Figure 5D,E show the frozen hydrogels absorbing 2 mmol L^−1^ of SS solution without a hydrate formation reaction. It was found that the frozen hydrogels looked smoother than hydrogels that experienced hydrate formation, suggesting that the rough areas might be hydrates. The region inside the yellow line is a whole hydrogel particle (Figure 5A). Hydrates not only emerged in pores inside of hydrogels (Figure 5B), but also appeared outside the hydrogel particle (Figure 5C), demonstrating that hydrate formation occurred inside and outside the hydrogels simultaneously. Specifically, a few smooth areas also existed inside the polymer networks, implying that some inner water was still unconverted to hydrates. This part of residual water was tightly absorbed by hydrogels and did not participate in hydrate formation, causing a lower water-to-hydrate conversion in HSS-2 (72.16 ± 2.07%) compared to that of SS-5 (86.63 ± 2.97%). Consequently, the storage capacity of the HSS-2 group was a little inferior to the SS-5 group.

### 2.3. Recycling Performance

**Figure 6:**
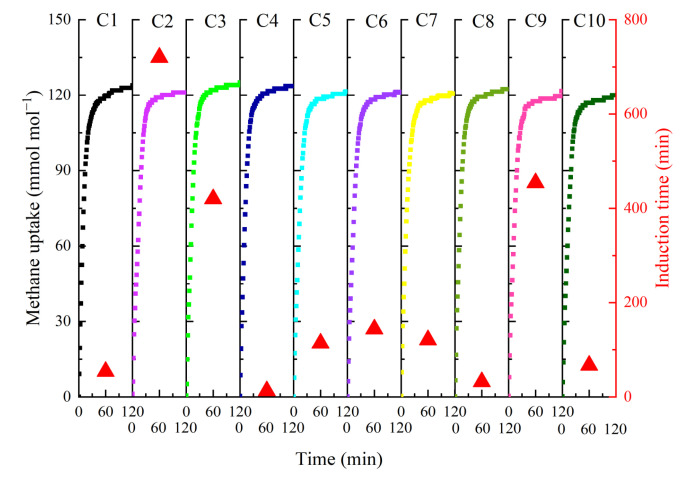
Methane uptakes (indicated by scatter curves) and induction times (represented by a red triangle) of 10 repeated hydrate formation–dissociation cycles promoted by HSS-2.

The hydrogel-SS hybrid medium exhibited good recoverable performance under observation of morphological evolutions of hydrates, which could re-absorb the released water from hydrate dissociation and recover its original shape and volume, inferring that this hybrid medium might perhaps hold admirable recyclability. Figure 6 depicts the methane hydrate formation kinetics during 10 repeated hydrate formation–dissociation cycles (C1 to C10) in the HSS-2 system. The trends for methane uptake curves in ten cycles were similar to each other, and along with the recycling time, the final methane uptake value did not decline and maintained at a narrow range of 119.97 to 125.06 mmol mol^−1^. As listed in Table 4, the HGR_St90_ of 10 cycles also did not change dramatically, and varied in a small range of just 0.203 to 0.241 mmol mL^−1^ min^−1^; and the induction periods were shorter than 150 min, except for C2, C3 and C9, which kept length values of 720, 420 and 454 min, respectively. Apparently, the recycling time of the hydrogel-SS hybrid medium had a negligible effect on methane hydrate formation kinetics in at least 10 repeated hydrate formation–dissociation cycles. Comparatively, the 10 recycling uses of the hydrogel-SS hybrid medium are more than 2, 7 and 8 times obtained by PSS-co-AAm-water [26], PVA-co-PAA-water [28] and PVA-co-PAA-SDS [29] hydrogel-based reaction media, respectively, confirming that it maintains a preferable recyclability.

Although the single use of PVA-co-PAA hydrogels or dilute SS solution (1–4 mmol L^−1^) has poor or even no promoting effects on methane hydrate formation kinetics, the hybrid medium composed by them can achieve an excellent synergistic promotion. According to the morphological observation of hydrate evolutions, hydrogels can greatly promote hydrate nucleation due to the large surface area, and SS can generate a strong capillary effect that induces rapid hydrate growth. Cryo-EM observation indicates that hydrates emerge inside as well as outside of hydrogel particles, inferring that hydrates can grow inward and outward simultaneously. Based on these results, the promotion mechanism can be speculated as follows: initially, hydrates nucleation tends to occur at the exposed outer surface of hydrogels, where methane molecules in headspace react with the free water on the hydrogel surface, and then hydrates grow along with the hydrogel surface and form as thin frosted shells totally covering the hydrogel surface; due to the existence of SS, these formed hydrate shells might possibly be porous, and can generate a strong capillary effect and drive some inner water to move out of hydrogels to participate in continuous outward hydrate growth, which completely encases hydrogel particles and even climbs along the reactor sidewall; and afterwards, these removed water molecules leave behind some temporary cavities inside of hydrogels, and methane gas can diffuse into these spaces and react with other water remaining in hydrogels, leading to continuous inward hydrate growth; as the hydrate shells continually thicken, the methane cannot diffuse into hydrogels anymore and the residue water can no longer transfer out of hydrogels, and as a result, the hydrate formation consequently finishes. During this process, hydrogels are primarily responsible for providing sufficient nucleation sites and enlarging the gas–liquid interface, while dilute SS is mainly in charge of producing the capillary effect, and they together contribute to improving mass transfer and synergistically promote hydrate growth.

This hybrid medium has high promotion efficiency, outstanding recyclability and satisfactory biodegradability, and is accordingly appropriate to be a packing medium for portage NGH tanks. However, it also suffers from a drawback, which is the relatively lower water-to-hydrate conversion compared to that of surfactants. The highest water-to-hydrate conversion of all tested groups in this work is merely 73.17 ± 1.32%. The poor water-to-hydrate conversion might be owed to some unconverted water inside the hydrogels. One part of unconverted water is non-freezing water, which is an inherent type of water absorbed by hydrogels that tightly links to polymeric chains though hydrogen bonding. The non-freezing water cannot be frozen at extremely low temperatures, and reasonably cannot be converted to hydrates [32]. Another part of unconverted water is the residue water that stably remains inside of hydrogels and cannot contact methane gas, which also cannot be converted to hydrates. These unconverted water molecules fail to form hydrate cages, consequently resulting in a slightly inferior methane storage capacity. In this regard, increasing mass transfer is essential for improving water-to-hydrate conversion, and some possible strategies are suggested as follows: (i) intermittent stirring can be applied in the reaction process in a hybrid medium to disperse hydrogel particles and alleviate hydrogel agglomeration, which can further enhance the gas–liquid interface and improve mass transfer; (ii) trying to introduce porous materials with different sizes (e.g., carbon nanotubes, graphene and active carbon) into hydrogels may be feasible, because it can manufacture sufficient hydrophobic pores or channels in hydrogels, which can provide hydrophobic micro spaces for methane accommodation in the hydrogel interior and improve mass transfer; (iii) a 3D printed lining jacket with specific structures (e.g., perforated in walls, honeycomb briquette-shaped or space-partitioned) can be added into the conventional hydrate reactor, which can act as a scaffold for hydrogels and increase the contact area between hydrogels and methane gas, giving rise to an enhanced mass transfer.

## 3. Conclusions

A hybrid reaction medium consisting of biodegradable hydrogels (PVA-co-PAA) and dilute SS solution (1–4 mmol L^−1^) was employed for hydrate-based methane storage. In this binary system, hydrogel particles with a large surface area could provide sufficient nucleation sites for hydrate formation and increase mass transfer, and at the same time, the dilute SS solution generated a strong capillary effect that further enhanced mass transfer; thus, their combination achieved a wonderful synergistic promotion of methane hydrate formation kinetics. The hybrid medium, containing 3 mmol L^−1^ of SS at a swelling ratio of 120 g g^−1^, attained the highest methane storage capacity of 122.6 ± 1.9 *v*/*v*. Additionally, the morphology observation of hydrate evolutions revealed that the hybrid medium could relieve the wall-climbing growth of hydrates promoted by bulk SS solution and improve hydrate compactness. More importantly, the hydrogel-SS hybrid medium showed excellent recyclability, and could keep efficient and stable promoting effects for at least 10 repeated hydrate formation–dissociation cycles. Accordingly, this hybrid reaction medium is effective and eco-friendly, is suitable as a packing medium for NGH storage tanks and shows great application prospects in hydrate-based methane storage.

## 4. Materials and Methods

### 4.1. Materials

Polyvinyl alcohol (98.0–99.0%) and sodium p-styrenesulfonate (≥90.0%) were supplied by Shanghai Aladdin Reagent Co., Ltd., Shanghai, China. Acrylic acid (≥99.0%); sodium hydroxide (≥96.0%) and ammonium persulfate (≥98.0%) were purchased from Sinopharm Chemical Reagent Co., Ltd., Shanghai, China. *N*,*N*-dimethylacrylamide (A.R.) was purchased from Shanghai McLean Biochemical Technology Co., Ltd., Shanghai, China. Methane (≥99.999%) was purchased from Qingdao Dehai Weiye Technology Co., Ltd., Qingdao, China. DI water was self-made in the laboratory (Ultra pure water machine, UPR-5TNZP, Sichuan Youpu Ultra Pure Technology Co., Ltd, Sichuan, China). All chemicals were used without further purification.

### 4.2. Methane Hydrate Formation in Hydrogel-SS Hybrid Medium

Biodegradable PVA-co-PAA hydrogels were prepared by the reported method [29]. The absorption capacity (*AC*) of hydrogels was measured in triplicate using free swelling methods. We submerged 0.05 g of dried hydrogels in 500 mL of liquid at room temperature for 48 h to reach swelling equilibrium. The swollen samples were fetched out and weighed (electronic balance, SQP, Sadolis Scientific Instruments Co., Ltd., Guangzhou, China). ACs of hydrogels on distilled water, 0.5, 1, 2, 3, 4 and 5 mmol L^−1^ of SS solutions were calculated using Equation (1) and are listed in Table 2:

(1) $$AC\left(g\;g^{-1}\right)=\frac{W_{S}-W_{d}}{W_{d}}$$

where *AC* is the absorption capacity in grams of water per gram of the dried hydrogel samples, $W_{d}$ and $W_{s}$ are the weights of dried and swollen hydrogels, respectively.

A stainless steel vessel with a volume of 100 mL and maximum pressure capability of 16 MPa was used as a reactor (high pressure reactor, 316L-100mL, Cheng Scientific Research Instrument Co., Ltd., Taoyuan City, Taiwan) for methane hydrate formation. Six reaction groups called SS-0.5, SS-1, SS-2, SS-3, SS-4 and SS-5 were produced in bulk SS solutions at concentrations of 0.5, 1, 2, 3, 4 and 5 mmol L^−1^, respectively. The other six reaction groups named HSS-0.5, HSS-1, HSS-2, HSS-3, HSS-4 and HSS-5 were performed in hydrogel-SS hybrid reaction media (at a unified swelling ratio of 120 g g^−1^), which employed hydrogels to absorb 0.5, 1, 2, 3, 4 and 5 mmol L^−1^ of SS solutions, respectively. Additionally, a control group only containing distilled water was also tested. All of the experimental scenarios are presented in Table 5, and each scenario was carried out in triplicate. After adding feedstocks, the vessel was sealed up and put in a cold bath (Constant temperature water bath, TMS8032-R05, Zhejiang Tomos Technology Co., Ltd., Hangzhou, China) at a constant temperature of 274.15 K. The vessel was flushed by methane gas (>99.9999%) three times and then charged by methane, reaching a pressure of 7 MPa. The pressure and temperature inside of the reactor were periodically detected by built-in sensors, respectively.

Induction time was the duration from the time point which pressure reached 7 MPa to the time point which pressure began to drop. Gas consumption (*n_t_*), methane uptake ($n_{M,t}$), water-to-hydrate conversion (*α*), hydrate growth rate (*HGR_t_*) and methane storage capacity of hydrates (SC) at time *t* during the methane hydrate formation process were calculated by Equations (2)–(7):

(2) $$n_{t}=\frac{\frac{P_{0}V_{0}}{Z_{0}RT_{0}}-\frac{P_{t}V_{0}}{Z_{t}RT_{t}}}{1-\frac{P_{t}∆Vm}{Z_{t}RT_{t}}}$$

(3) $$Z_{t}=1+\left[0.083-0.422\times {\left(\frac{T_{c}}{T_{t}}\right)}^{1.6}\right]\frac{P_{t}T_{c}}{P_{c}T_{t}}+\omega \left[0.139-0.172\times {\left(\frac{T_{c}}{T_{t}}\right)}^{4.2}\right]\frac{P_{t}T_{c}}{P_{c}T_{t}}$$

(4) $$n_{M,t}=\left(M_{w}n_{t}\right)/m_{w}$$

(5) $$\alpha =\left(6.0M_{w}n_{t}\right)/m_{w}$$

(6) $$HGR_{t}=\frac{n_{t}}{V_{w}\times t}$$

(7) $$c_{S}=n_{t}\times \frac{V_{mg}\times V_{mw}}{V_{w}\times \left(V_{mw}+∆V\right)}$$

where *P* and *T* are the pressure and temperature in the reactor, respectively; *V* is the volume of the gas phase in the reactor; *R* is the universal gas constant; m is the hydration number; Δ*V* is the molar volume difference between methane hydrates and water; *Z* is the compressibility factor; *T_c_*, *P_c_* and ω are 190.6 K, 4.599 MPa and 0.012 for methane, respectively; *M_w_* is the molar weight of water; *m_w_* is the initial mass of water in the sample; *V_w_* is the volume of the initial reaction solution; *V_mg_* and *V_mw_* are the molar volumes of gas and water, respectively; the subscripts 0 and *t* are the time during HFP.

### 4.3. Morphology Observation of Hydrate Formation and Dissociation

#### 4.3.1. Stacked Hydrogels Absorbing Dilute SS

A borosilicate glass reactor with a volume of 15 mL and maximum pressure capability of 10 MPa served as a reactor for visualized methane hydrate formation. The temperature of the glass reactor was constantly maintained at 274.15 K by a water bath jacket totally coating the reactor. Individually, 2 and 5 mmol L^−1^ of SS solution were pre-dyed by red dye powder. Together, 1 mL of colored SDS solution and 0.0083 g of dried hydrogels were placed in the bottom of the glass reactor. After 2 h to achieve full swelling, the reactor was sealed off and flushed by methane gas three times, and then charged by methane until 7 MPa. The pressure and temperature during the hydrate formation process were measured by sensors. After hydrate formation finished, the pressure was reduced to the atmospheric pressure to enable hydrates to dissociate completely. The morphological evolutions of hydrates during formation and dissociation processes were observed by an optical microscope (GP-300C, Precision Instrument Co., Ltd., Kunshan Gaopin, Zhejiang, China) and recorded by a computer. In addition, hydrate formation and dissociation processes in 2 or 5 mmol L^−1^ of bulk SS solution were also observed. The height increment of hydrates at the end of the hydrate formation process was measured. A parameter named the elongation coefficient (*EC*), which was defined as the ratio of the hydrate height increase to the water-to-hydrate conversion, was used to reflect the hydrate compactness in different reaction systems and was calculated by Equation (8):

(8) $$EC=\frac{h_{t}-h_{0}}{h_{0}\alpha }$$

where *h_t_* is the hydrate height at time *t* during HFP; *h_0_* is the initial height of the reaction system; *α* is the water-to-hydrate conversion at time *t* during HFP.

#### 4.3.2. Single-Grained Hydrogel Particle Absorbing Dilute SS

One hydrogel particle (355–600 μm) was fully swelled by 2 or 5 mmol L^−1^ of SS solution at a swelling ratio of 120 g g^−1^ and then placed on a stainless steel plate. The plate was horizontally placed in the glass reactor. The reactor was sealed off, and the reaction and observation procedures complied with the procedures for stacked hydrogels mentioned above. Additionally, hydrate formation and dissociation processes in a droplet (distilled water, 2 or 5 mmol L^−1^ of SS solution) with a volume of 40 μL were also observed.

### 4.4. Cryo-Electron Microscopy Observation

Hydrate morphology and distribution in hydrogel networks were performed with a cryo-electron microscope (Cryo-EM, SN-3400, HITACHI, Wollerau, Switzerland). Hydrates formed in the HSS-2 group were collected and observed by Cryo-EM. As for the control group, a frozen hydrogel swelled by 2 mmol L^−1^ of SS solution was also observed by Cryo-EM.

### 4.5. Recyclability Tests

The recycling performance of HSS-2 was investigated in the stainless steel vessel. When the first cycle of hydrate formation finished, the vessel was depressurized to the atmospheric pressure, and then it was taken out of the water bath and kept quiescent for 4 h at a temperature of 298.15 K. After the hydrates dissociated completely, the vessel was again placed in a water bath at a temperature of 274.15 K, and methane gas was again charged into the reactor to 7 MPa, and the second cycle started. Additional cycles were accomplished by repeating the above procedures.

## Figures and Tables

**Table 1:** Methane hydrate formation kinetics in different reaction systems.

Group	Induction Time (min)	HGR_t90_ (mmol mL^−1^ min^−1^)	Methane Uptake (mmol mol^−1^)	Storage Capacity (*v*/*v*)
DI water	Undetected	-	-	-
SS-0.5	Undetected	-	-	-
SS-1	Undetected	-	-	-
SS-2	Undetected	-	-	-
SS-3	Undetected	-	-	-
SS-4	Undetected	-	-	-
SS-5	115.8 ± 203.8	0.380 ± 0.075	144.4 ± 4.9	143.1 ± 4.9
HSS-0.5	Undetected	-	-	-
HSS-1	11.3 ± 16.3	0.184 ± 0.006	122.3 ± 1.6	121.2 ± 1.6
HSS-2	18.7 ± 32.3	0.250 ± 0.004	122.6 ± 3.1	121.5 ± 3.1
HSS-3	216.0 ± 332.6	0.296 ± 0.053	123.7 ± 1.9	122.6 ± 1.9
HSS-4	410.0 ± 420.4	0.292 ± 0.021	121.7 ± 1.6	120.6 ± 1.6
HSS-5	625.3 ± 653.2	0.306 ± 0.023	120.1 ± 1.8	119.1 ± 1.8

**Table 2:** ACs of PVA-co-PAA hydrogels on distilled water and different concentrations of SS solutions. (The data for distilled water were cited from ref. [29]).

Liquid Type	AC (g g^−1^)
Distilled water	330.7 ± 8.4
0.5 mmol L^−1^ of SS solution	292.5 ± 4.8
1 mmol L^−1^ of SS solution	251.9 ± 6.2
2 mmol L^−1^ of SS solution	203.9 ± 2.3
3 mmol L^−1^ of SS solution	189.3 ± 3.1
4 mmol L^−1^ of SS solution	175.5 ± 5.6
5 mmol L^−1^ of SS solution	151.1 ± 9.7

**Table 3:** Hydrate height increments and growth ECs in SS-5 and HSS-2 groups.

Reaction System	Hydrate Height Increment (mm)	EC
SS-5	16.1 ± 0.9	1.847
HSS-2	8.2 ± 1.4	1.109

**Table 4:** Methane hydrate formation kinetics in HSS-2 system in 10 repeated hydrate formation–dissociation cycles.

Number of Cycles	Induction Time (min)	HGR_t90_ (mmol mL^−1^ min^−1^)	Methane Uptake (mmol mol^−1^)	Storage Capacity (*v*/*v*)
C1	54	0.229	123.92	122.82
C2	720	0.219	121.67	120.00
C3	420	0.223	125.06	123.96
C4	13	0.241	123.64	122.55
C5	114	0.215	121.50	120.43
C6	144	0.205	120.98	119.91
C7	121	0.218	120.61	119.54
C8	32	0.229	122.43	121.34
C9	454	0.203	121.62	120.54
C10	67	0.215	119.97	118.91

**Table 5:** Experiment scenarios of methane hydrate formation in stainless steel vessel.

Group	Hydrogel	Liquid
DI water	-	10 mL of distilled water
SS-0.5	0	10 mL of 0.5 mmol L^−1^ SS solution
SS-1	0	10 mL of 1 mmol L^−1^ SS solution
SS-2	0	10 mL of 2 mmol L^−1^ SS solution
SS-3	0	10 mL of 3 mmol L^−1^ SS solution
SS-4	0	10 mL of 4 mmol L^−1^ SS solution
SS-5	0	10 mL of 5 mmol L^−1^ SS solution
HSS-0.5	0.0833 g	10 mL of 1 mmol L^−1^ SS solution
HSS-1	0.0833 g	10 mL of 2 mmol L^−1^ SS solution
HSS-2	0.0833 g	10 mL of 3 mmol L^−1^ SS solution
HSS-3	0.0833 g	10 mL of 4 mmol L^−1^ SS solution
HSS-4	0.0833 g	10 mL of 5 mmol L^−1^ SS solution
HSS-5	0.0833 g	10 mL of 1 mmol L^−1^ SS solution

## Data Availability

The data presented in this study are openly available in the article.
